# Elevated fibrous sheath interacting protein 1 levels are associated with poor prognosis in non-small cell lung cancer patients

**DOI:** 10.18632/oncotarget.14575

**Published:** 2017-01-10

**Authors:** Yuqiang Mao, Ran Xu, Xiaoying Liu, Wenjun Shi, Yun Han

**Affiliations:** ^1^ Department of Thoracic Surgery, Shengjing Hospital of China Medical University, Shenyang 110004, China; ^2^ Department of Plastic Surgery, The First Hospital of China Medical University, Shenyang 110001, China

**Keywords:** fibrous sheath interacting protein 1, non-small cell lung cancer, prognosis, TNM staging

## Abstract

In this study, we examined the expression and prognostic value of fibrous sheath interacting protein 1 (FSIP1) in 202 non-small cell lung cancer (NSCLC) patients who underwent lung cancer resection at Shengjing Hospital of China Medical University. FSIP1 mRNA and protein expression were measured in NSCLC tissues and non-tumor adjacent tissues (NATs), and Harrell's concordance index (c-index) was used to evaluate the ability of FSIP1 to predict prognosis. FSIP1 mRNA and protein expression was higher in NSCLC tissues than in NATs. Survival analysis revealed the 5-year overall survival rate to be 35.4% in the FSIP1-positive group and 56.3% in the FSIP1-negative group, and FSIP1-positive status was an independent prognostic factor for poor overall survival. The c-index value of FSIP1 for overall survival was greater than that of Ki67, and the addition of FSIP1 status increased the c-index value of the TNM staging system. These results suggest that evaluating FSIP1 status in addition to TNM stage during routine pathological examinations could improve prognostic predictions in NSCLC patients.

## INTRODUCTION

Lung cancer is one of the most frequently diagnosed cancers and the leading cause of cancer-related death in China [1]. Although considerable advances have been made in surgery, adjuvant chemoradiotherapy, and targeted therapy, the prognosis for lung cancer remains poor [2]. This high mortality rate may be partly due to the lack of effective prognostic biomarkers. Currently, prognostic predictions are largely based on TNM staging. However, lung cancer patients at the same TNM stage may have different prognoses. New prognostic biomarkers are needed to more accurately identify high-risk lung cancer patients with poor prognoses.

Fibrous sheath interacting protein 1 (FSIP1) is a component of the microtubule- and dynein-rich fibrous sheath structure, which is necessary for flagellum function and sperm movement [3]. FSIP1 mRNA expression, which is low or undetectable in most normal tissues, is elevated in breast tumors [4]. *Zhang et al*. [5] reported that FSIP1 protein levels are also elevated in breast cancer tissues, and higher levels were associated with poorer prognosis in breast cancer patients. However, the role of FSIP1 in lung cancer remains unknown.

In this study, we measured FSIP1 expression in non-small cell lung cancer (NSCLC) and analyzed the association between FSIP1 and clinicopathological features. In addition, we evaluated the prognostic value of FSIP1 to determine whether it might be useful as a supplementary biomarker together with TNM stage in NSCLC patients.

## RESULTS

### FSIP1 expression is elevated in NSCLC

**Figure 1:**
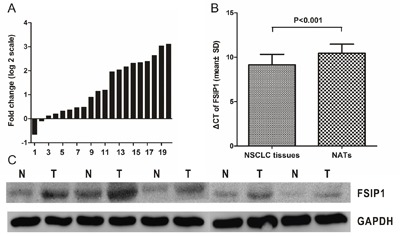
FSIP1 mRNA and protein expression are elevated in NSCLC **A**. Data are presented as log_2_ of fold-change in FSIP1 in NSCLC tissues relative to non-tumor adjacent tissues. Each case was analyzed in triplicate and repeated three times. **B**. ΔCT values were used to compare the relative expression of FSIP1 in NSCLC tissues and non-tumor adjacent tissues. Data are shown as means ± SD. Larger ΔCT values indicate lower expression. **C**. Western blot indicated that FSIP1 protein was overexpressed in NSCLC tissues compared to non-tumor adjacent tissues.

FSIP1 was measured in 20 NSCLC tissues and non-tumor adjacent tissues (NATs) pairs using real-time PCR. FSIP1 expression was higher in NSCLC tissues compared to NATs in 90% (18/20) of these pairs (Figure 1A), and FSIP1 expression was significantly higher in NSCLC tissues than in NATs (*p*<0.001, Figure 1B). Similar results were obtained when protein levels were examined using western blots (Figure 1C).

**Figure 2:**
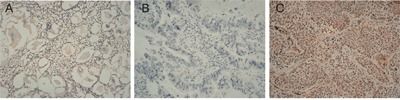
Immunohistochemical staining for FSIP1 in NSCLC tissues and non-tumor adjacent tissues. Magnification ×200 **A**. Non-tumor adjacent tissue (no stain). **B**. Negative FSIP1 staining in NSCLC tissues. **C**. Positive FSIP1 staining in NSCLC tissues.

We then used immunohistochemistry to measure FSIP1 protein expression in 202 tissue pairs; 54% (109/202) of these NSCLC tissues were FSIP1-positive and 46% (93/202) were FSIP1-negative. In squamous carcinoma, FSIP1 expression was predominantly in tumor cell nucleus and also appearing in the cytoplasm. While in adenocarcinoma, FSIP1 expression was predominantly in the cytoplasm (Figure 2). Additionally, FSIP1 expression was higher in NSCLC tissues than in NATs (IS, 6.021 ± 2.805 vs. 4.050 ± 2.586, respectively, *p*<0.001, Supplementary Table 1).

### Correlations between FSIP1 status and clinicopathological features

We also examined associations between FSIP1 expression and clinicopathological features using the chi-square test. FSIP1-positive status was correlated with more advanced TNM stages (*p*=0.042) and tended to be associated with more advanced pN categories (*p*=0.066, Table 1), although the latter association did not reach statistical significance. However, no significant relationships were found between FSIP1 expression and other clinicopathological features such as gender, age, histologic type, differentiation, and pT category (all *p*>0.05, Table 1).

**Table 1:** Association between FSIP1 status and clinicopathological features

Variables	Number (%)	FSIP1 status		*P* value
		FSIP1 (−) (%)	FSIP1 (+) (%)	
Sample size	202 (100)	93 (46.0)	109 (54.0)	
Gender				0.175
Male	96 (47.5)	49 (52.7)	47 (43.1)	
Female	106 (52.5)	44 (47.3)	62 (56.9)	
Age(y)				0.608
>60	119 (58.9)	53 (57.0)	66 (60.6)	
≤60	83 (41.1)	40 (43.0)	43 (39.4)	
Histologic type				0.144
AC	153 (75.7)	66 (71.0)	87 (79.8)	
SC	49 (24.3)	27 (29.0)	22 (20.2)	
Differentiation				0.710
Well	90 (44.6)	39 (41.9)	51 (46.8)	
Moderate	79 (39.1)	37 (39.8)	42 (38.5)	
Poor	33 (16.3)	17 (18.3)	16 (14.7)	
pT category				0.104
T1	82 (40.6)	40 (43.0)	42 (38.5)	
T2	89 (44.1)	45 (48.4)	44 (40.4)	
T3	18 (8.9)	5 (5.40)	13 (11.9)	
T4	13 (6.4)	3 (3.20)	10 (9.2)	
pN category				0.066
N0	129 (63.9)	67 (72.0)	62 (56.9)	
N1	22 (10.9)	9 (9.7)	13 (11.9)	
N2	50 (24.8)	16 (17.2)	34 (31.2)	
N3	1 (0.5)	1 (1.1)	0 (0)	
Distant metastasis				0.223
Negative	194 (96.0)	91 (97.8)	103 (94.5)	
Positive	8 (4.0)	2 (2.2)	6 (5.5)	
TNM stage				0.042
I	96 (47.5)	49 (52.7)	47 (43.1)	
II	45 (22.3)	25 (26.9)	20 (18.3)	
III	53 (26.2)	17 (18.3)	36 (33.0)	
IV	8 (4.0)	2 (2.2)	6 (5.5)	

Abbreviation, AC: adenocarcinoma; FSIP1: fibrous sheath interacting protein 1; SC: squamous carcinoma.

### FSIP1 expression is associated with poor prognosis

**Figure 3:**
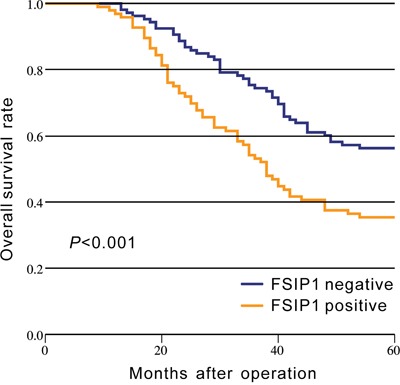
Kaplan-Meier analysis of overall survival based on FSIP1 status in 202 NSCLC patients The 5-year overall survival rate in the FSIP1-positive group was lower than that in FSIP1-negative group (*p*<0.001).

The 5-year overall survival (OS) rate was 35.4% in the FSIP1-positive patient group and 56.3% in the FSIP1-negative group (*p*<0.001, Figure 3). Furthermore, Cox multivariate analysis revealed that FSIP1-positive status was an independent prognostic factor for poor OS (HR = 1.876, 95% CI = 1.274–2.764, *p* = 0.001, Table 2).

**Table 2:** Univariate and multivariate analyses of overall survival in NSCLC patients

Variable	Univariate		Multivariate	
	HR (95% CI)	*P*	HR (95% CI)	*P*
Gender		0.537		
Female	1.000			
Male	1.120 (0.781-1.605)			
Age (y)		0.174		
≤60	1.000			
>60	1.297 (0.892-1.885)			
Histologic type		0.084		
AC	1.000			
SC	0.669 (0.424-1.055)			
Differentiation		0.486		
Well	1.000			
Moderate	1.256 (0.846-1.863)			
Poor	1.237 (0.736-2.078)			
pT category		<0.001		0.038
T1-T2	1.000		1.000	
T3-T4	2.198 (1.411-3.424)		1.624 (1.028-2.563)	
pN category		<0.001		0.006
Negative	1.000		1.000	
Positive	2.253 (1.556-3.240)		1.726 (1.171-2.543)	
Distant metastasis		<0.001		<0.001
Negative	1.000		1.000	
Positive	6.583 (3.158-13.725)		3.920 (1.829-8.400)	
TNM stage		<0.001		
I-II	1.000			
III-IV	3.847 (2.503-5.913)			
FSIP status		<0.001		0.001
Negative	1.000		1.000	
Positive	2.176 (1.489-3.180)		1.876 (1.274-2.764)	
Ki67 status		0.006		0.019
Negative	1.000		1.000	
Positive	1.769 (1.179-2.654)		1.629 (1.082-2.453)	

Abbreviation, AC: adenocarcinoma; FSIP1: fibrous sheath interacting protein 1; SC: squamous carcinoma.

We also examined the relationship between Ki67 expression status and prognosis. The 5-year OS rate was 39.2% in the Ki67-positive group and 59.2% in the Ki67-negative group (*p*=0.004, Supplementary Figure 1). Cox multivariate analysis also indicated that Ki67-positive status was an independent prognostic factor for poor OS (HR = 1.629, 95% CI = 1.082–2.453*, p* = 0.019, Table 2).

### PFSIP1 has a higher prognostic ability

**Figure 4:**
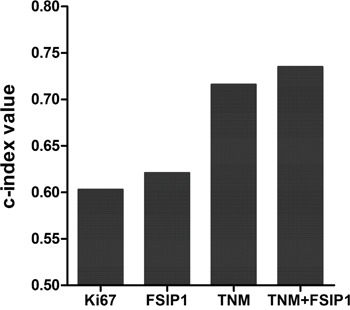
Comparison of c-index values for Ki67, FSIP1, TNM stage, and TNM+FSIP1

We used the c-index method to evaluate the prognostic ability of different models. The c-index value of FSIP1 was greater than that of Ki67 (0.621 vs. 0.603), and the c-index value was greater for TNM and FSIP1 together than for TNM staging alone (0.735 vs. 0.716, Figure 4).

## DISCUSSION

Here, we measured FSIP1 expression in tissues from NSCLC patients. FSIP1 mRNA and protein expression were both higher in NSCLC tissues than NATs. We also found that FSIP1-positive status was correlated with more advanced TNM stages and poorer prognosis. In addition, FSIP1-positive status was an independent prognostic factor for poor OS. To our best of knowledge, this is the first study to explore the role of FSIP1 in NSCLC.

FSIP1 is a component of the microtubule and dynein-rich fibrous sheath structure and may directly or indirectly support cell mitosis [3]. Indeed, Cappell *et al*. reported that FSIP1 depletion can enhance paclitaxel-induced mitotic arrest and/or the formation of micronucleated cells in NSCLC cell lines, and FSIP1-mediated alterations in microtubule and dynein function may support the microtubule network and enhance mitotic robustness in cancer cells [3]. In addition, FSIP1 can bind to and activate cancer/testis antigen proteins (including CABYR, SPA17, AKAP3, AKAP4, and ROPN1) in the fibrous sheath in tumor cells, in turn promoting cancer progression [3, 6–8]. These results are consistent with the association observed here between FSIP1-positive status and more advanced TNM stages and poorer prognosis in NSCLC. However, additional studies of the molecular mechanisms underlying the role of FSIP1 in NSCLC are required.

Ki67 levels, which are correlated with cancer cell proliferation and growth, are widely used in routine pathological examinations as a proliferation marker [9, 10]. In addition, Ki67 is also used as a prognostic and diagnostic index for the evaluation of cancer biopsies, including lung cancer [11, 12]. Our results confirmed that Ki67 was an independent prognostic factor in NSCLC (Table 2). We also used the c-index method to compare the prognostic capacities of Ki67 and FSIP1. The c-index value of FSIP1 was greater than that of Ki67, suggesting that FSIP1 had better prognostic capacity than Ki67. FSIP1 might therefore be particularly valuable during routine pathological examinations in NSCLC patients. However, it is worth noting that this study included only 202 NSCLC patients from a single institution; multicenter, large-scale studies are needed to confirm our results in NSCLC patients more generally. Additionally, due to limited data availability, we were not able to analyze the association between FSIP1 and the efficacy of adjuvant therapy in NSCLC; future studies are needed to evaluate that relationship as well.

We also compared the prognostic ability of FSIP1 in combination with the TNM staging system to the ability of the TNM staging system alone. The c-index for OS was greater for TNM+FSIP1 than for TNM staging alone, indicating that the addition of FSIP1 status improved the prognostic ability of the TNM staging system. Thus, FSIP1 may increase prognostic accuracy in NSCLC patients and might serve as a valuable supplementary index when used with the current TNM staging system.

In conclusion, we found that FSIP1 was highly expressed in NSCLC and was an independent prognostic factor in NSCLC patients. These results suggest that the evaluation of FSIP1 in combination with the current TNM staging system during routine examinations might help improve prognostic predictions in NSCLC patients.

## MATERIALS AND METHODS

### Patients and samples

Primary NSCLC tissues and paired non-tumor adjacent tissues (NATs) were obtained from 202 patients who underwent lung cancer resection at Shengjing Hospital of China Medical University (Shenyang, China) between November 2009 and October 2013. Of these samples, 20 NSCLC tissues and paired NATs were assayed for FSIP1 mRNA and protein expression using real-time reverse transcription polymerase chain reaction (real-time PCR) and western blot, respectively. In addition, all 202 sample pairs were used for immunohistochemistry and included in prognosis analysis. Follow-up times ranged from 3 to 83 months with a median of 48 months. Tumor grades were staged according to 7th edition of the TNM staging system. The study was approved by the Research Ethics Committee of China Medical University. Written informed consent was obtained from all patients.

### RNA extraction, reverse transcription, and real-time PCR

Total RNA was isolated from tissue samples using TRIzol reagent (Invitrogen Life Technologies, USA). Reverse transcription was conducted using the PrimeScript™ RT reagent Kit with gDNA Eraser (Takara, China). Real-time PCR analyses were performed using SYBR Premix Ex Taq™ (Takara, China) on a Light Cycler 480 II Real-Time PCR system (Roche Diagnostics, Switzerland). The following primers were used: FSIP1, 5'-GTGTTCCCCCAGCTTTCCA-3' (forward) and 5'-TGCTTCAGTGACAAGAGCTTC-3' (reverse); GAPDH, 5'-CGGATTTGGTCGTATTGGG-3' (forward) and 5'-CTGGAAGATGGTGATGGGATT-3' (reverse). Relative FSIP1 expression was normalized to the GAPDH reference and calculated using the 2^–ΔΔ CT^ method [13].

### Western blots

Total protein was isolated using a protein extraction kit (ProMab, USA) followed by centrifugation. The protein content was quantitatively analyzed using a bicinchoninic acid protein assay and separated using 12% SDS-PAGE (sodium dodecyl sulfate polyacrylamide gel electrophoresis). The separated proteins were transferred to a PVDF (polyvinylidene fluoride) membrane (Millipore, USA). Samples were blocked in 5% bovine serum albumin for 2 h at room temperature and incubated with anti-FSIP1 (1:1000; Novus, USA) and anti-GAPDH (1:10000; Sigma, USA) primary antibodies as appropriate overnight at 4°C. The membrane was washed in PBST and incubated with horseradish peroxidase-conjugated goat anti-rabbit IgG at room temperature for 1 h. The membrane was washed with PBST again and the ECL kit was used for western blot detection. Relative FSIP1 protein levels compared to the GAPDH control were determined using ImageJ.

### Immunohistochemistry

PV-9000 Polymer Detection System Immuno-Histological Staining (Zhongshan, Beijing, China) was used for immunostaining. Four-μm-thick NSCLC tissue and NATs sections were obtained using a cryostat, deparaffinized with xylene, and rehydrated using a graduated ethanol series. In order to block endogenous peroxidase activity, tissue sections were initially incubated with 0.3% hydrogen peroxide solution for 10 min. Sections were then incubated for 60 min at room temperature with rabbit polyclonal FSIP1 antibody (1:200 dilution, Novus, USA) and washed with phosphate buffered saline (PBS). Sections were then incubated with Polymer Helper at room temperature for 20 min and washed with PBS. Secondary polyperoxidase-antibody was then added and incubated at room temperature for 30 min, followed by a final PBS wash. Diaminobenzidine was used as a chromogen to visualize staining. Negative control staining, in which the primary antibody was omitted, was conducted in parallel.

### Evaluation of immunohistochemical staining results

The immunostaining results were evaluated by two pathologists independently using a semi-quantitative scoring system. Staining intensity values (0 for no staining; 1 for weak straining; 2 for moderate straining; and 3 for strong straining), and values representing the percentage of cells stained (0 for ≤5%; 1 for 5-25%; 2 for 25-50%; 3 for 50-75%; and 4 for ≥75%) were assigned. These staining intensity and positive cell percentage values were multiplied to generate immunoreactivity scores (IS) [14]. Any scoring discrepancies were resolved by discussion. All tissue samples were then assigned to one of two groups based on IS; FSIP1-positive status was defined by detectable nuclear/cytoplasm immunoreactivity and an IS ≥ 4 as determined by a receiver operating characteristic curve.

### Statistical analysis

Continuous variables were analyzed using paired *t*-tests or non-parametric tests. Categorical variables were examined using the chi-square test. Survival rate was determined using the Kaplan-Meier method with log-rank tests. Univariate analysis was used to explore associations between prognostic factors and prognosis. Significant prognostic factors in univariate analysis were then analyzed in multivariate analyses using the Cox proportional hazards model.

The predictive capacity of different models was evaluated by measuring discrimination, which is the ability to distinguish between high-risk and low-risk patients. Discrimination was quantified using Harrell's concordance index (c-index) [15, 16]. A c-index value of 1.0 indicates a perfect discrimination; c-index values closer to 1.0 indicate more accurate predictive ability.

All data were analyzed using SPSS software version 20.0 and STATA software version 12.0. A two-tailed *p*-value < 0.05 was considered statistically significant.

### Ethic statement

The study was approved by the Research Ethics Committee of China Medical University. Written informed consent was obtained from all patients.

## SUPPLEMENTARY MATERIALS FIGURES AND TABLES


