# Supplementary material for: Elevated fibrous sheath interacting protein 1 levels are associated with poor prognosis in non-small cell lung cancer patients
Source: Oncotarget. 2017 Jan 10;8(7):12186–93. doi: 10.18632/oncotarget.14575 (PMC5355335; doi:10.18632/oncotarget.14575)
Supplement: Supplementary file 1 [file oncotarget-08-12186-s001.pdf]

Elevated fibrous sheath interacting protein 1 levels are associated with poor prognosis in non-small cell lung cancer patients

SUPPLEMENTARY FIGURE AND TABLE

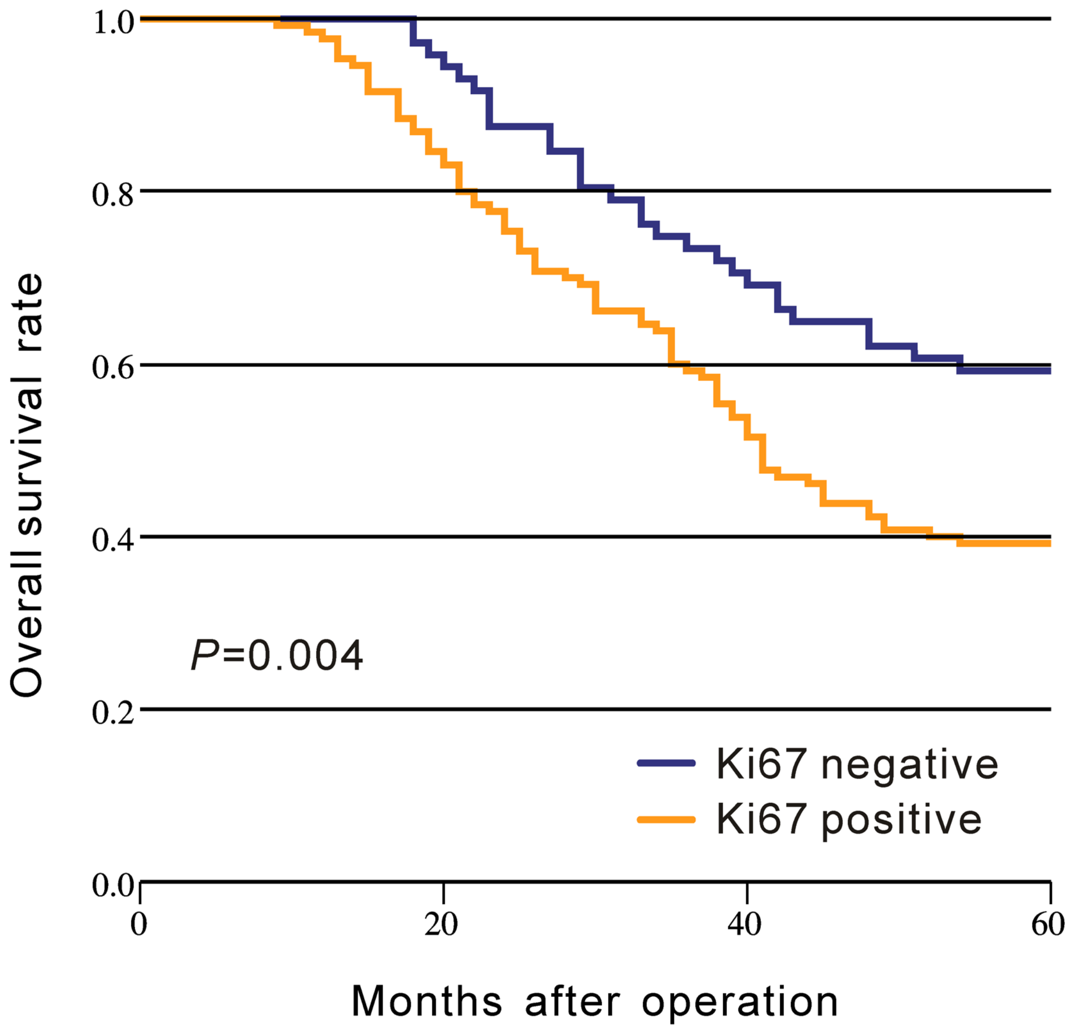

Supplementary Figure 1: Kaplan-Meier analysis of overall survival based on Ki67 status in 202 NSCLC patients. The 5-year overall survival rate in the Ki67-positive group was lower than that in Ki67-negative group (*p*=0.004).

Supplementary Table 1: Immunoreactivity score of tissues

Tissues	No.	IS (Mean ± SD)	*P*
NSCLC	109	6.021 ± 2.805	<0.001
NAT	93	4.050 ± 2.586	

Abbreviation, IS: immunoreactivity score; NAT: non-tumor adjacent tissue; NSCLC: non-small cell lung cancer; SD: standard deviation.
